# New computed tomographic predictors of complicated perioperative course of 17.5F mini-percutaneous nephrolithotomy (mini-PNL)

**DOI:** 10.1007/s00345-020-03388-5

**Published:** 2020-08-09

**Authors:** Tomasz Ozimek, Jan M. Laturnus, Carolin Gohlke, Judith R. Wiessmeyer, Julian P. Struck, Marie C. Hupe, Axel S. Merseburger, Mario W. Kramer

**Affiliations:** Department of Urology, University Hospital Schleswig-Holstein, Campus Lübeck, Ratzeburger Allee 160, 23562 Lübeck, Germany

**Keywords:** Percutaneous nephrolithotomy, Renal stones, Complications, Computed tomography, Access angle, T12LP

## Abstract

**Purpose:**

Radiological parameters predicting a postoperative stone-free status (SFS) or a complicated perioperative course of mini-PNL, are scarce. Our aim was to identify such factors for prone 17.5F mini-PNL.

**Methods:**

A monocentric cohort of 103 cases was retrospectively analysed for factors predicting SFS and relevant complications, i.e. Clavien–Dindo (CD) ≥ 2. Parameters measured on preoperative supine CT included maximal stone diameter, skin-to-stone distance (SSD), ideal tract length (ITL), access angle, minimal T12—Lower Kidney Pole distance (T12LP) and minimal Iliac Crest—Lower Kidney Pole distance (ICLP). Infundibulopelvic angle (IPA) was measured on intraoperative pyelography.

**Results:**

The median maximal stone diameter was lower in cases with postoperative SFS [16 mm (Min. 10; Max. 35) vs. 20 mm (Min. 6; Max. 85), *p *= 0.0052]. CD ≥ 2 was more frequent in cases with a bigger stone burden [19 mm (Min. 13; Max. 85) vs. 16 mm (Min. 6; Max. 49), *p *= 0.0056] and with the ribs in the access angle [7/23 (30.43%) vs. 8/76 (10.53%); *p *= 0.0454]. T12LP significantly differed in cases with and without CD ≥ 2 [80.48 mm (± 21.31) vs. 90.43 mm (± 19.42), *p *= 0.0397]; however, it had no influence on SFS (*p* > 0.05). SSD, ITL, IPA and ICLP were significant regarding neither SFS nor CD ≥ 2 prevalence (*p* > 0.05). Using multivariate logistic regression, T12LP was confirmed as an independent predictor on CD ≥ 2 prevalence.

**Conclusions:**

Preoperative computed tomographic factors indicating elevated kidney position influence perioperative course of mini-PNL. T12LP and the presence of ribs in the access angle are, apart from stone diameter, the most useful indicators for cases at risk of CD ≥ 2.

## Introduction

Percutaneous nephrolithotomy is the preferred option for the treatment of large kidney stones > 2 cm in diameter. Due to its miniaturization, it is also considered a viable approach for a smaller stone burden [1]. Despite the wide choice of devices for percutaneous stone treatment (4.8–24F), mini-PNL is preferred in most clinical settings, as a compromise between good outcomes, safety and acceptable operation times [2]. However, the procedure is associated, as every endourologic procedure is, with substantial perioperative complications [3], postoperative urinary tract infections or sepsis [4]. To identify high-risk cases and to increase patient’s safety, several risk factors, such as staghorn stones, positive preoperative urine culture, diabetes, patient’s age, prolonged operation time or upper pole access, have already been determined [4–6].

Apart from stone burden itself, other radiological parameters predicting a postoperative stone-free status (SFS) or complicated perioperative course of percutaneous procedures are scarce. Thus, our aim was to identify such factors, based on preoperative computed tomography (CT) in supine position, and intraoperative pyelographic imaging, for prone 17.5F mini-PNL.

## Material and methods

A monocentric cohort of patients scheduled for prone 17.5F mini-PNL (103 cases) in our department between 2014 and 2019 was retrospectively analysed for factors predicting postoperative SFS and relevant perioperative complications Clavien–Dindo (CD) ≥ 2.

Written patient consent was obtained at least 24 h preoperatively. Microbiological testing was performed less than 2 weeks preoperatively. No routine preoperative antibiotic prophylaxis was applied. In patients with positive preoperative urine culture, a preoperative antibiotics course was administered and continued postoperatively. For patients with negative urine cultures, antibiotic prophylaxis, usually fluoroquinolones (ciprofloxacin), was administered intraoperatively, and continued orally during the postoperative hospital stay. All patients were operated under general anaesthesia in a classic prone position. Mini-PNL tract was dilated for the 17.5F working channel and surgery was performed with a 12F MIP M nephroscope (Karl Storz, Germany). The hospital stay for an uncomplicated postoperative course was limited to 72 h.

Stone-free status was, in the majority of the cases, determined intraoperatively by an endourologist. A radiological postoperative re-evaluation with a non-contrast CT was not a standard and was indicated only in case of uncertainty regarding postoperative stone-free status or prior to second-look procedure. A recurrent stone former was defined as a patient with at least one stone episode in the past.

**Fig. 1:**
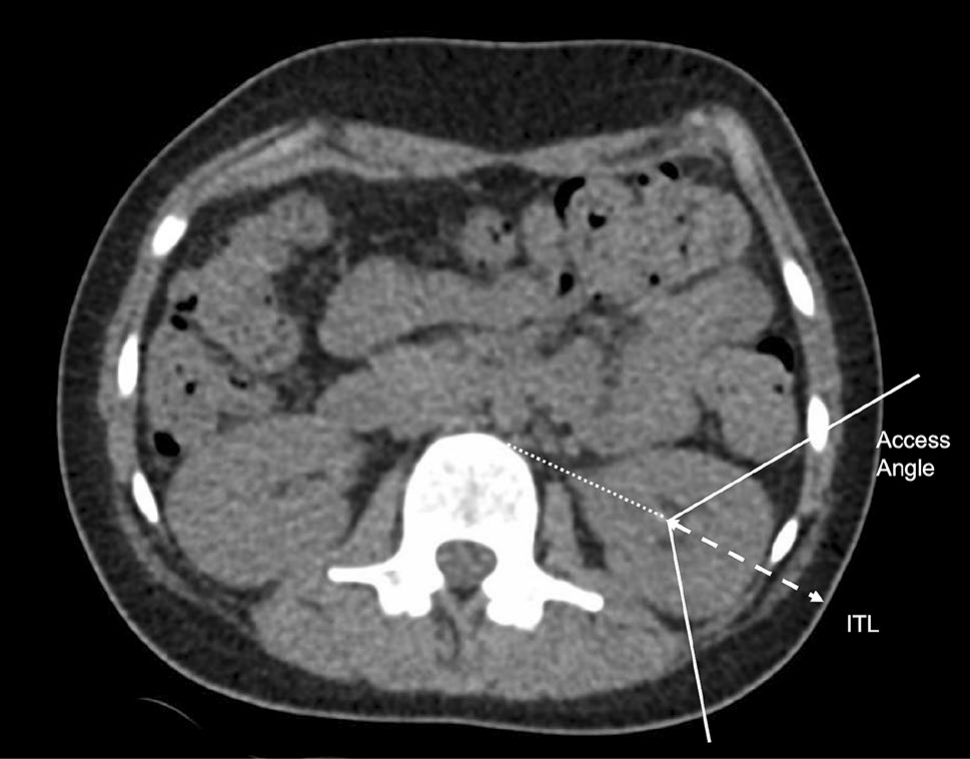
Ideal tract length (ITL) with the presence of ribs in the access angle measured on axial CT images

**Fig. 2:**
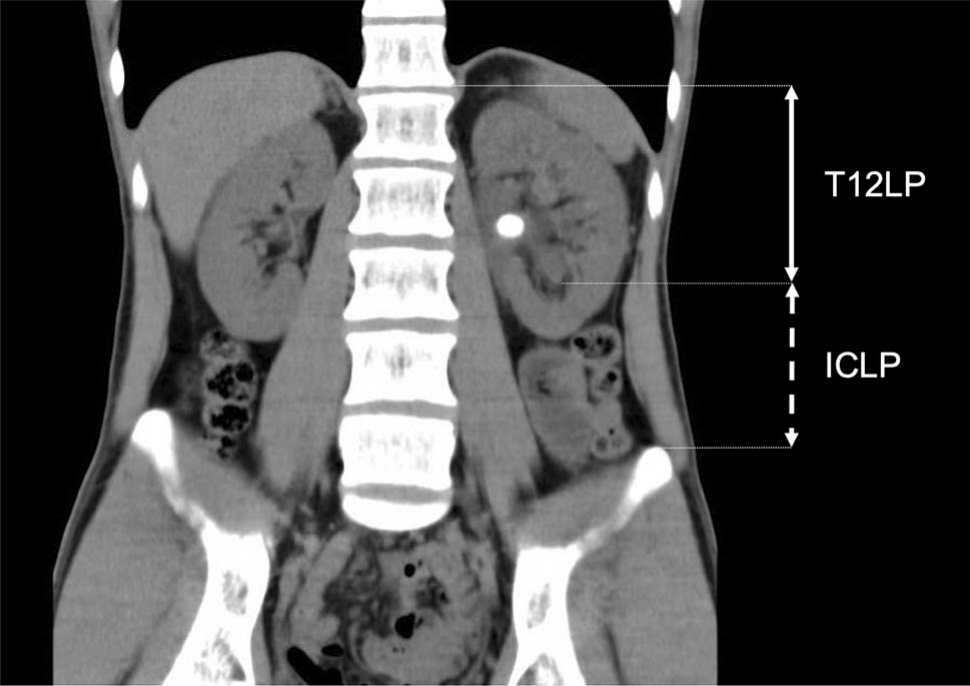
Minimal distance between T12 vertebra and lower calyx (T12LP) and between lower calyx and iliac crest (ICLP) measured on coronal CT images

Parameters were measured using Agfa HealthCare IMPAX Software and were based on preoperative CT in the supine position. These parameters included maximal stone diameter, skin-to-stone distance (SSD), ideal tract length (ITL), access angle, minimal T12—Lower Kidney Pole distance (T12LP) and minimal Iliac Crest—Lower Kidney Pole distance (ICLP). Measurements were taken on native CT scans; however, a urographic phase was additionally taken into consideration for better identification of calyceal anatomy, whenever available (7/103 cases). SSD was calculated as the arithmetic mean of three distances from the middle of the dominant stone to the skin, at 0º, 45º and 90º on axial CT images [7]. According to Marchini et al. [8] ITL was determined as the distance between the bottom of the lower calyx and the skin, on an imaginary line from the antero-lateral edge of the vertebra, through the bottom of the posterior calyx to the skin (Fig. 1). An access angle was drawn on axial CT images, with the vertex in the bottom of the punctured calyx, one leg adjacent to the lateral circumference of the paraspinous muscles and the other at the first adjacent organ [8] (Fig. 1). We also used coronal CT images to determine the minimal vertical distance between the bottom of the lower calyx and bone structures as the lowest point of T12 vertebra (T12LP), and the most cranial aspect of the iliac crest (ICLP) (Fig. 2). The infundibulopelvic angle (IPA) was retrospectively measured in accordance with the El-Bahnasy [9], and based on recorded intraoperative retrograde pyelography (RPG) images. The angle was determined between the ureteropelvic axis and the central axis of the lower pole infundibulum. The Clavien–Dindo scale was applied to classify the intra- and post-operative complications [10].

Uni- and multi-variate statistical analyses were performed in RStudio (version 1.0.136). Mean values with standard deviation (SD) for normally distributed variables, median values with minimum and maximum values for variables without normal distribution and percent values for categorical variables were used for descriptive statistics. The Chi-square test was applied for qualitative data whenever applicable. Normal distribution of quantitative data was proven with the Shapiro–Wilk test. The Mann–Whitney *U* (MWU) test or unpaired *t* test was conducted for univariate analysis. The level of statistical significance was *p* < 0.05.

## Results

We retrospectively analysed a cohort of 103 patients scheduled for 17.5F mini-PNL in our department, between 2014 and 2019. Working channel was successfully established in 92.23% (95/103) of cases. The access was placed supracostal in one case. The general cohort and stone characteristics are presented in Table 1. Overall ipsilateral stone-free rate was 73.79% (76/103) for median maximal stone diameter of 17.0 mm (Min 6.0; Max. 85.0 mm). The CD ≥ 2 complication rate was 22.33% (23/103), and constituted mostly of postoperative febrile infections (18/23; 78.26%), that were defined as systemic inflammatory response syndrome (SIRS), requiring additional postoperative intravenous antibiotic therapy. In 66.67% (12/18) of cases, this prolonged the hospital stay over the standard 72 h. Non-infectious complications were associated with postoperative macrohaematuria (2 patients), neurological complications (transient ischemic attack––1 patient; postoperative syncope––1 patient) and pneumonia (1 patient). In five cases (5/23; 21.74%) with CD ≥ 3A, severe complications, either ureteral catheter placement or intensive care, were postoperatively required. No complications leading to death were recorded.

**Table 1:** Univariate analysis of general and stone characteristics regarding stone-free status and complications

	Overall	Stone-free status				Clavien–Dindo ≥ 2			
		Yes	No	*p* value	Test	Yes	No	*p* value	Test
Length of stay (days)	3 (1; 15)	3 (2; 9)	3 (1; 15)	0.2184	MWU	5 (2; 15)	3 (1; 10)	0.0001	MWU
Gender (male/female)	60/43	41/35	19/8	0.2079	Chi2	9/14	51/29	0.0615	Chi2
Age (years)	56.68 (SD 15.14)	59 (23; 84)	54.59 (SD 14.25)	0.2953	MWU	56.78 (SD 16.35)	56.65 (SD 14.88)	0.9707	*t* test
Side (right/left)	43/60	32/44	11/16	1	Chi2	13/10	30/50	0.1644	Chi2
BMI (kg/m^2^)	28 (14; 51)	28.71 (SD 6.53)	28.63 (SD 7.66)	0.958	*t* test	38.26 (SD 6.81)	27 (14; 51)	0.1411	MWU
ASA > 2 (yes/no)^a^	35/65	29/45	6/20	0.214	Chi2	8/15	27/50	1	Chi2
Previous PNL (yes/no)	4/99	1/75	3/24	0.0924	Chi2	0/23	4/76	0.6301	Chi2
Previous SWL (yes/no)	7/96	7/69	0/27	0.2347	Chi2	2/21	5/75	1	Chi2
Previous URS (yes/no)	5/98	4/72	1/26	1	Chi2	1/22	4/76	1	Chi2
Recurrent stone former (yes/no)	42/61	31/45	11/16	1	Chi2	10/13	32/48	0.9534	Chi2
Positive urine culture (yes/no)	43/60	31/45	12/15	0.9174	Chi2	15/8	28/52	0.0188	Chi2
Prestenting (yes/no)	77/26	56/20	21/6	0.8707	Chi2	19/4	58/22	0.477	Chi2
Nephrostomy Preoperatively (yes/no)	4/99	3/73	1/26	1	Chi2	1/22	3/77	1	Chi2
Guy’s stone score (1/2/3/4)^b^	40/30/17/11	36/22/9/5	4/8/8/6	0.0024	Fisher exact	7/6/3/7	33/24/14/4	0.01991	Fisher exact
Hounsfield units	906 (SD 336)	895 (SD 333)	943 (SD 354)	0.6418	*t* test	917 (SD 281)	902 (SD 358)	0.882	*t* test
Radiopacity (yes/no)^3^	89/12	66/9	23/3	1	Chi2	22/1	67/11	0.366	Chi2
Concomitant ureterolithiasis (yes/no)	15/88	11/65	4/23	1	Chi2	5/18	10/70	0.4403	Chi2
Lower pole stone (yes/no)	72/31	50/26	22/5	0.1996	Chi2	15/8	57/23	0.7657	Chi2
Lower pole stone—maximal diameter (mm)	13.05 (SD 4.77)	13.11 (SD 4.78)	12.86 (SD 4.90)	0.8645	*t* test	15 (6; 19)	12.83 (SD 4.97)	0.3494	MWU
Dominant stone position (lower/middle/upper pole/pelvis)	34/4/3/62	29/2/2/43	5/2/1/19	0.1537	Fisher exact	5/2/1/15	29/2/2/47	0.2357	Fisher exact
Dominant stone—maximal diameter (mm)	17 (6; 85)	16 (10; 35)	20 (6; 85)	0.0052	MWU	19 (13; 85)	16 (6; 49)	0.0056	MWU
Number of affected calyceal groups	1 (0; 3)	1 (0; 3)	2 (0; 3)	0.0054	MWU	1 (0; 3)	1 (0; 3)	0.9626	MWU
Number of relevant kidney stones (1/2/3/ > 3)	58/19/6/20	48/13/4/11	10/6/2/9	0.0711	Fisher exact	11/4/2/6	47/15/4/14	0.6211	Fisher exact
Predominantly calcium oxalate stone composition (yes/no)	51/37	38/32	13/5	0.2682	Chi2	9/10	42/27	0.4276	Chi2

Standard deviation for mean values and minimum/maximum for median values in brackets

^a^No data in 3 cases, ^b^no data in 5 cases, ^c^no data in 2 cases

As expected, the median values of maximal stone diameter were significantly lower in cases with postoperative SFS [16 mm (Min. 10; Max. 35) vs. 20 mm (Min. 6; Max. 85), *p *= 0.0052]. The incidence of relevant complications, CD ≥ 2, was more frequent in cases with a bigger stone burden [19 mm (Min. 13; Max. 85) vs. 16 mm (Min. 6; Max. 49), *p *= 0.0056] and with the ribs in the access angle [7/23 (30.43%) vs. 8/76 (10.53%); *p *= 0.0454]. T12LP significantly differed in cases with and without CD ≥ 2 complications [80.48 mm (SD 21.31) vs. 90.43 mm (SD 19.42), *p *= 0.0397]; however, it had no influence on SFS (*p* > 0.05). SSD, ITL, IPA and ICLP parameters were not predictors for postoperative SFS or CD ≥ 2 prevalence (*p* > 0.05). Guy’s Stone Score was confirmed as relevant for SFS (*p *= 0.002) and CD ≥ 2 (*p *= 0.0199) prediction.

Radiologic measurements and perioperative characteristics are presented in Table 2. Using multivariate logistic regression for continuous radiological data, T12LP was confirmed as an independent predictor for CD ≥ 2 complications, with a moderate power of discrimination––area under the curve = 0.65. Short T12LP, defined as < 90 mm, was measured in 18 out of 23 cases (78.26%), with CD ≥ 2 complications. The specificity for the prediction of CD ≥ 2 complications, for cases with ribs in the access angle on preoperative CT, was 89.47% (sensitivity 30.43%, positive predictive value 46.67% and negative predictive value 80.95%).

**Table 2:** Univariate analysis of radiological and perioperative characteristics regarding stone-free status and complications

	Overall	Stone-free status				Clavien–Dindo ≥ 2			
		Yes	No	*p* value	Test	Yes	No	*p* value	Test
Stone to skin distance (mm)^a^	101.7 (SD 28.72)	103.0 (SD 27.15)	97.72 (SD 33.07)	0.4284	*t* test	102.9 (SD 29.51)	101.3 (SD 28.66)	0.8177	*t* test
Ideal tract length (mm)^a^	100.1 (SD 28.26)	101.5 (SD 28.37)	96.37 (SD 30.34)	0.4349	*t* test	105.8 (SD 29.35)	98.37 (SD 28.68)	0.2826	*t* test
T12-lower pole distance (mm)^a^	88.07 (SD 20.29)	89.51 (SD 20.04)	84.15 (SD 20.85)	0.2519	*t* test	80.48 (SD 21.31)	90.43 (SD 19.42)	0.0397	*t* test
Iliac crest-lower pole distance (mm)^a^	44.04 (SD 20.06)	41.62 (SD 19.41)	50.41 (SD 20.71)	0.0522	*t* test	51.04 (SD 22.20)	41.89 (SD 19.00)	0.0552	*t* test
Access angle—window of puncture (°)^a^	87.42 (SD 24.35)	88.82 (SD 25.10)	83.7 (SD 22.24)	0.3544	*t* test	81.83 (SD 21.58)	89.12 (SD 25.01)	0.2098	*t* test
Ribs in the access angle (yes/no)^a^	15/84	8/64	7/20	0.1295	Chi2	7/16	8/68	0.0454	Chi2
Infundibulopelvic angle (°)	55.29 (SD 16.74)	55.67 (SD 16.45)	54.05 (SD 18.01)	0.6919	*t* test	51.3 (SD 19.80)	56.57 (SD 15.58)	0.1906	*t* test
DJ postoperatively (yes/no)	101/2	74/2	27/0	0.9686	Chi2	22/1	79/1	0.927	Chi2
Nephrostomy postoperatively (yes/no)	18/85	8/68	10/17	0.0048	Chi2	6/17	12/68	0.3563	Chi2
Lower pole puncture successful (yes/no)	91/3	70/1	21/2	0.2958	Chi2	20/0	71/3	0.8428	Chi2
At least one unsuccessful puncture (yes/no)**	25/76	17/58	8/18	0.5746	Chi2	6/17	19/59	1	Chi2
Any puncture successful (yes/no)^b^	99/2	75/0	24/2	0.1076	Chi2	23/0	76/2	1	Chi2
Real supracostal puncture (yes/no)	1/102	1/75	0/27	1	Chi2	0/23	1/79	1	Chi2
Lower pole access successful (yes/no)^c^	85/10	69/1	16/9	0.0001	Chi2	18/3	67/7	0.8156	Chi2
Any access successful (yes/no)^b^	93/8	75/0	18/8	0.0001	Chi2	21/2	72/6	1	Chi2
Number of accesses	1(0; 2)	1(1; 1)	1 (0; 2)	0.0001	MWU	1(0; 1)	1(0; 2)	0.734	MWU
combined with URS (yes/no)	26/77	19/57	7/20	1	Chi2	8/15	18/62	0.3561	Chi2
Laser energy (kJ)	3.96 (0.11; 408.2)	3.46 (0.11; 408.2)	20.19 (SD 5.85	0.0053	MWU	6.55 (0.26; 408.2)	3.64 (0.11; 34.07)	0.1405	MWU
Fluoroscopy time (s)	242 (24; 758)	241 (24; 651)	246 (34; 758)	0.3522	MWU	225 (131; 584)	259.5 (24; 758)	0.8424	MWU
Operation time (min)	103.5 (35; 242)	98 (51; 199)	138.8 (SD 48.94)	0.0001	MWU	127.7 (SD 40.59)	99 (35; 242)	0.01338	MWU
Clavien–Dindo ≥ 2 (yes/no)	23/80	13/64	10/16	0.0413	Chi2				
Postoperative UTI (yes/no)	18/85	10/66	8/19	0.1008	Chi2				

Standard deviation for mean values and minimum/maximum for median values in brackets

^a^No data in 4 cases, ^b^nephrostomy used for a tract dilation in 2 cases, ^c^nephrostomy used for a tract dilation in 1 case

## Discussion

According to data from the CROES study group, the prone position is being preferred in approximately 80% of cases for percutaneous stone surgery [11]. Preoperative radiological diagnostics is an important tool for the planning percutaneous access, and identifying eventual conflicts of the possible tract with adjacent organs (e.g. spleen, liver and colon) as a cause of major complications [8, 12].

Our study presents two new radiological CT-based predictive factors for a complicated perioperative course that can be measured on every CT, but do not depend on a possible conflict of the puncture tract with adjacent internal organs. Neither T12LP nor the presence of ribs in the access angle has been studied so far. Our results support the hypothesis that the elevated retroperitoneal position of the kidney, indicated by a reduced T12LP, and the position of the lower pole behind the costal margin with the ribs in the access angle may complicate the perioperative course due to a more challenging access for the mini-PNL.

It is unclear whether computed tomographic preoperative imaging in the spinal position is suitable enough for mini-PNL in the prone position. Marchini et al. [8] suggested that due to an elevated risk of organ injury while aiming at the upper pole in the prone position, exact ITL should be preoperatively planned, based on CT images in the prone position. In our opinion, supine CT is also sufficient for preparation before prone surgery, on the condition that intraoperative ultrasound is applied for improved recognition of potential conflicts with adjacent structures, while establishing the working channel. In our department, combined sonography and fluoroscopy is an intraoperative standard. Kidney puncture is sonographic-guided and is followed by fluoroscopic-guided tract dilatation. Using this technique, no organ injuries were recorded in the analysed cohort.

Further studies would be helpful to prove, if CT in the prone position could possibly provide more reliable information regarding presented parameters, and by this mean facilitate prone percutaneous surgery. In our opinion, CT in the prone position, instead of the standard spinal position, should be discussed with the radiologist, as a diagnostic tool for an acute flank pain, especially with sonographic stone signal.

Our study did not entirely cover factors influencing SFS and associated complications, however, indicates possible directions for future research.

The anatomically elevated position of the kidney, expressed in reduced T12LP or the presence of ribs in the access angle, is according to our results associated with a complicated perioperative course of mini-PNL. Thus, we advise a special attention to the preoperative body positioning. An additional tilting of the head and legs away from the operated side could increase the T12LP and facilitate the percutaneous access. The effects of such modification and the role of parameters as T12LP in other body positions for mini-PNL should, however, be confirmed in further studies.

The literature on radiological factors predicting outcomes of mini-PNL is limited. To increase the accuracy of SFS prediction in the preoperative setting, numerous tools, such as Guy’s Stone Score, S.T.O.N.E. Score or CROES Score, have been developed [13, 14]. For SFS prediction, we confirmed the role of stone size and numbers of affected calyceal groups as crucial parameters, already included in aforementioned scores. Other parameters in the S.T.O.N.E score including ITL, stone density and preoperative hydronephrosis, had in our study no influence on SFS prediction. In our opinion, further research to establish scores or nomograms for the prediction not only of SFS but also of complication risks are warranted.

The retrospective character is the main drawback to our study. Intraoperative and postoperative antibiotic prophylaxis was always administered, as recommended by EAU and AUA guidelines [1, 15]. However, no standard policy for patients with negative urine cultures was applied. The choice of antibiotic type and dose in this subgroup was made intraoperatively by the operating surgeon. To exclude any potential influences of bias regarding the prevalence of infectious complications, we suggest a detailed antibiotic algorithm for future prospective studies, validating our results.

Increased postoperative urinary tract infection (UTI) rates are known problems in patients with positive preoperative urine cultures [16]. Despite preoperative microbiological testing and targeted treatment at least 48 h prior to surgery, the complications rate was still significantly elevated among the analysed patients with positive urine cultures. Due to the retrospective nature of our study, we were unable to define whether a prolonged preoperative antibiotic administration would decrease postoperative UTI rates. In our opinion, further studies on this topic are required to determine optimal antibiotic regimens for the preoperative preparation of patients at risk from positive preoperative urine cultures.

In eight cases, a working channel could not be established, mostly due to an unsuccessful dilatation caused by hypermobile kidney (six of eight cases). This well-known phenomenon is associated with factors, including gender (female), lowered BMI and previous open stone surgery [17]. No radiological predictors of renal displacement have yet been determined and would be an interesting topic for the future research.

IPA is a parameter of special interest due to its ability to predict complications, as well as a flexible ureteroscope damage risk during flexible ureteroscopy (fURS) [18]. During mini-PNL, the ureter plays anatomically a less important role when compared to a fURS procedure. We showed that contrary to fURS, IPA had no influence on the outcomes and complications of mini-PNL. Thus, a percutaneous approach could surely be considered as an option, especially for cases with steep IPA.

Our data on the gender-associated prevalence of UTIs are consistent with the literature and indicate a higher trend rate for infectious complications after percutaneous surgery in females [19]. This observation supports the notion that special attention to antibiotic prophylaxis and sufficient perioperative antibiotic therapy should focus especially on this group.

## Conclusions

Not only stone characteristics but also preoperative radiologic factors indicating anatomically elevated position of the kidney influence the perioperative course of mini-PNL. Apart from stone diameter, T12LP and the presence of ribs in the access angle appear to be the most useful regarding computed tomographic preoperative indications of cases at risk of CD ≥ 2. SSD and ITL predict neither SFS nor the occurrence of CD ≥ 2 complications.
